# Environmental Exposure to Pesticides and the Risk of Child Neurodevelopmental Disorders

**DOI:** 10.3390/medicina60030475

**Published:** 2024-03-13

**Authors:** Rocio Parrón-Carrillo, Bruno José Nievas-Soriano, Tesifón Parrón-Carreño, David Lozano-Paniagua, Rubén Trigueros

**Affiliations:** 1 Department of Psychology, Faculty of Health Sciences, University of Almeria, 04120 Almeria, Spain; rpc349@inlumine.ual.es (R.P.-C.); rtr088@ual.es (R.T.); 2 Department of Nursing, Physiotherapy and Medicine, Faculty of Health Sciences, University of Almeria, 04120 Almeria, Spain; tpc468@ual.es

**Keywords:** pesticides, environmental exposure, neurodevelopment, learning disorders, children

## Abstract

*Background and Objectives*: Neurodevelopment is a fragile brain process necessary for learning from the beginning of childhood to adulthood. During the procedure, several risks could affect it, including environmental factors such as neurotoxic chemicals or environmental pollutants and, within them, exposure to pesticides. *Materials and Methods*: This ecological descriptive study attempted to assess the association between environmental exposure to pesticides and neurodevelopmental disorders. This study was conducted on 4830 children diagnosed for 11 years in a total population of 119,897 children in three areas: high, medium, and low greenhouse concentrations. *Results*: Chromosomal abnormalities were the most common prenatal disorder (28.6%), while intrauterine physical factors were the least common (0.5%). Among perinatal diagnoses, gestational age less than 32 weeks was the most common (25%), while hyperbilirubinemia requiring exchange transfusion and birth complications was the least common (0.4%). Brain damage was the most common problem detected in postnatal diagnosis (36.7%), while unspecified postnatal abnormalities were the least common (3.1%). *Conclusions*: The areas with the highest greenhouse concentration had higher incidences of neurodevelopmental disorders, particularly in boys, and lower age of referral. Chromosomal abnormalities were prevalent for prenatal diagnoses, gestational age below thirty-two weeks for perinatal diagnoses, and brain damage for postnatal diagnoses. Future studies should analyze the connection between pesticide exposure and neurodevelopmental disorders using spatial point pattern analysis.

## 1. Introduction

Neurodevelopment is the main brain process for learning [1]. Brain development is a complex process that begins early and continues beyond birth and leads to the maturation of structures, the acquisition of skills, and the formation of the individual as a unique person [2]. The crucial periods for normal brain development are intrauterine life (prenatal and perinatal stages) and the first weeks of life (postnatal stage). The early stages of the nervous system’s formation start with the ectoderm, one of the germ layers. The neural crest is formed from this layer and continues to develop the peripheral and central nervous systems [3,4,5,6]. Neuronal system development begins in the first 3–4 weeks of gestation and represents a crucial stage in neurobiological development. 

Maternal exposure to xenobiotic substances, including pesticides, during brain organogenesis and histogenesis (prenatal and perinatal stages) can alter the neurodevelopment of the fetus and, consequently, of the newborn. Furthermore, newborns can be exposed to xenobiotics through inhalation, absorbing pesticides in their environment, breastfeeding, or feeding infant formula during the postnatal period, altering brain maturation. These alterations could lead to lesions and functional alterations detected in later phases, even in adulthood. Early intervention programs aim to implement measures before the onset of any neurodevelopmental disorder by identifying prenatal, perinatal, and postnatal risk factors within biological, familial, and social dimensions. The Diagnostic Organization for Early Intervention (ODAT) framework was developed to streamline existing criteria, describing a three-tier structure across multiple axes: biological (axis 1), family (axis 2), and environmental (axis 3) risk factors. This study is focused on the biological risk factor axis, where the risk factors that the ODAT defines as associated with each stage are analyzed [7].

Neurodevelopmental disorders can have a profound impact on schoolchildren, with conditions such as intellectual disability, attention deficit hyperactivity disorder, autism spectrum disorder, specific learning disorder, communication disorders, or motor disorders [8]. Specific learning disorders affect a significant percentage of children, ranging from 5 to 15%, according to the American Psychiatric Association [9]. A total of 8,309,575 students are enrolled in Spain’s non-university general education stages, consisting of 4,280,699 boys and 4,028,876 girls. Spain’s Ministry of Education and Vocational Training reported that 40,618 students enrolled in Special Education during the 2022/23 school year [10]. 

However, attention deficit hyperactivity disorder is the most common psychiatric disorder in childhood and adolescence [11]. According to the Spanish Federation of Associations for Attention Deficit and Hyperactivity Disorders, 5% of the population of children and adolescents has attention deficit hyperactivity disorder, equivalent to one or two children per classroom [12]. Regarding autism spectrum disorder, the World Health Organization (WHO) estimated in 2020 that 1 in 160 children worldwide has autism spectrum disorder [13]. However, the Centers for Disease Control and Prevention established an increasing trend for diagnosing autism spectrum disorders in some communities (1.85% of children aged 8 years) [14]. It suggests an epidemiological problem because the burden of these disorders on affected children, families, and communities is economic, emotional, and academic difficulties [15]. Global economic costs are unknown, but in Spain, it is estimated that 2017 special education spending was approximately EUR 1.35 billion [16].

Commonly used pesticides have been well documented to cause neurological damage through various mechanisms such as oxidative stress, neuroinflammation, elevated calcium levels within cells, neuronal cell death, and changes in neurotransmitter levels [17]. Some pesticides can cause hyperactivity, loss of vitality, poor coordination and memory, impaired drawing ability, delayed neurodevelopmental development, behavioral disorders, and motor problems due to exposure in the fetal and early postnatal periods [18]. Therefore, professionals who care for and educate children should understand the fragility of their development from childhood to adulthood. It includes the recognition of the multiple manifestations of neurodevelopmental peculiarities, such as gross and fine motor, sensory, language, and socio-emotional alterations [2]. Pesticides can also have complex neurotoxic effects and cause subtle changes that are not related to their impact on hormones. 

The prenatal, postnatal, and adolescent periods are critical windows for brain development and microbiota development. Exposure to certain pesticides during pregnancy could lead to neurodevelopmental disorders in children [19]. It could potentially affect memory, cognition, and linguistics and even increase the risk of autism spectrum disorder [20]. Some studies have found a positive dose–response association between breastfeeding, intelligence quotient, and school performance [21]. However, exposure of an infant to lipophilic pesticides (such as pyrethroids) or their metabolites through breast milk could have an adverse effect. This impact could be due to the direct and specific neurotoxic effect of pesticides on development.

Furthermore, exposure could trigger dysbiosis in the gut microbiota through the gut–microbiota–brain axis, increasing the predisposition to neurodevelopmental deficits [22,23,24]. Therefore, alteration of the intestinal microbiota due to exposure to pesticides during breastfeeding could also alter children’s neurodevelopment. Recent studies have shown that children with autism spectrum disorder who underwent microbiota transfer therapy had significant improvements in behavior [20]. Furthermore, a recent publication highlighted the role of epigenomics in contributing to autism spectrum disorder [25]. Exposure could disrupt some physiological processes and have adverse health effects, even affecting the human epigenetic profile [26].

Environmental exposure could affect neurodevelopmental disorders, including specific learning disorders, in children living in areas with a high concentration of greenhouses, where pesticides are widely used. Therefore, this study focuses on analyzing this aspect of neurodevelopmental disorders. This study aims to explore the prevalence of neurodevelopment disorders and their prenatal, perinatal, and postnatal risk factors in children living in areas with different levels of pesticide use, according to the age of access to the early intervention program and the sex.

## 2. Materials and Methods

### 2.1. Study Design

An ecological descriptive epidemiological study was conducted in Almeria, Southeastern Spain. Almeria, a province of Andalusia, is known to have the largest concentration of greenhouses in the world, covering 32,827 ha [27]. This study divided the region into three areas—West Almeria, Central Almeria, and East Almeria—based on different pesticide use according to agronomic criteria provided by the Andalusian Council of Agriculture and coincident with the three health districts [28,29,30]. The West Almeria district has the highest use of pesticides, with a cultivated area of 22,189 ha (67.6% of the entire study area) and an annual consumption of approximately 6352.08 tons of pesticides. The Central Almeria district has moderate pesticide use, with 9950 ha of greenhouses (30.3% of the study area) and an estimated pesticide consumption of 2698.57 tons. The East Almeria district has the lowest pesticide use, with 688 ha of plastic-covered land (2.1% of the entire study area) and a pesticide consumption of 209 tons. 

Almeria has gained global recognition for its research into environmental and occupational pesticide exposure due to the high concentration of greenhouses in the region. In particular, plastic-grown crops possess unique characteristics, such as high humidity and heat, which can significantly increase production levels but also promote the development of pests that require the use of pesticides. As this is a prevalence study in areas of high pesticide use, there is no differentiation in single- or multiple-pesticide-use areas. Therefore, several types of pesticides are used. According to previous studies conducted on the use of pesticides in greenhouses in this area, insecticides and fungicides have been described as the most used compounds. Specifically, macrocyclic lactones, neonicotinoids, pyrethroids, triazoles, anilinopyrimidines, and copper salts are the pesticides used the most frequently in the study area, including biopesticides [31].

### 2.2. Study Population

A comprehensive study was conducted on children referred to Early Intervention Centers of the Territorial Delegation of Health and Families in the Almeria region for 11 years (from 2011 to 2022). Regarding districts, Almeria Central has 48,823 children, representing 40.79% of the population. Of these children, 25,264 are boys and 23,559 are girls. East Almeria, conversely, comprises 21,557 children, representing 18.0% of the population. Among these children, there are 11,045 boys and 10,512 girls. Lastly, West Almeria has 49,517 children, representing 41.3% of the population. Of these children, 25,368 are boys and 24,149 are girls. All individuals were diagnosed with any neurodevelopmental disorder. The research included a sample size of 4830 children between 0 and 5 diagnosed with any pathology listed according to the ODAT criteria [7].

Variables analyzed in this study included sex, age at the time of referral, health district, and diagnosis. The remaining variables were the diagnoses reflected in the ODAT classification [32]. Data were previously anonymized. Diagnoses were always given by pediatric specialists who were part of the Early Care program, using protocolized criteria to minimize the possibility of error. This study was approved by the Research Ethics Committee of the University of Almeria (reference EFM 279/23) and agreed with the Declaration of Helsinki for International Health Research.

### 2.3. Statistical Analysis

This study analyzed categorical variables using frequencies and percentages. Quantitative variables were analyzed using central tendency (mean) and dispersion (standard deviations) measures. Additionally, after performing the Kolmogorov–Smirnov normality tests, a bivariate statistical analysis was performed using the Mann–Whitney U and Kruskal–Wallis tests to compare quantitative variables. Differences in diagnosis or sociodemographic factors according to exposure area were assessed using Pearson’s chi-square test. To determine the risk of learning disability, multiple binary logistic regression was performed, which was adjusted for health district, sex, and age at referral. These variables were considered because they could influence the statistical model. The confidence intervals were established at 95% and a significance level of *p* < 0.05 was used. Statistical analysis was performed with IBM SPSS Statistics for Windows, version 28.0 (Armonk, NY, USA).

## 3. Results

Table 1 summarizes the demographic characteristics of the study population. Boys had more medical conditions than girls. The average age of referral was 2.05 ± 1.38 years. The district with the highest gross percentage of referrals was Central Almeria (41.7%), and the lowest was the East district (20.1%).

**Figure 1:**
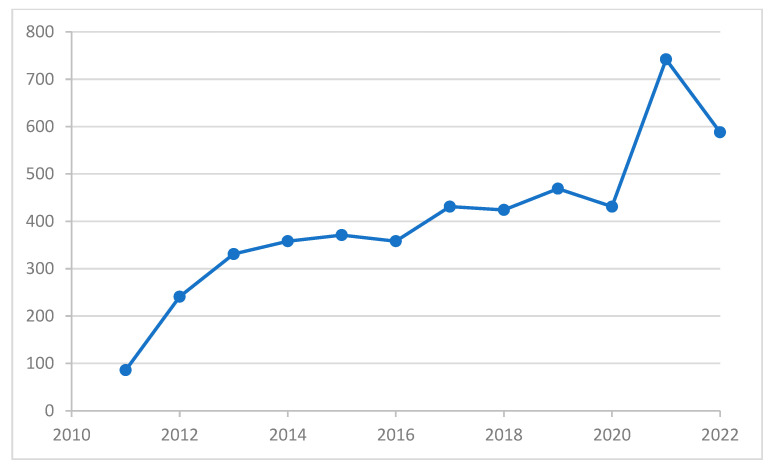
Distribution of the number of children with neurodevelopmental disorders diagnosed annually by the Early Attention Centers of the Territorial Delegation of Health and Families in the province of Almeria. X-axis: year of diagnosis; Y-axis: number of new referrals with neurodevelopmental disorders diagnosed by the Early Attention Centers of the Territorial Delegation of Health and Families.

The trend in the number of new referrals with neurodevelopmental disorders diagnosed annually by the Early Attention Centers of the Territorial Delegation of Health and Families in the province of Almeria over the 11 years of study, from 2011 to 2022, is presented in Figure 1. Although there is a slight decrease in referrals in 2022, the overall pattern indicates a growing trend (from 86 to 588). 

**Figure 2:**
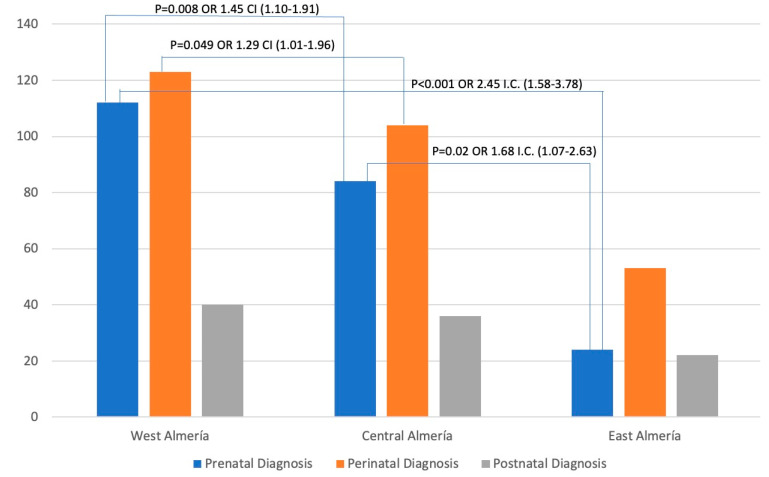
Cases diagnosed by district according to prenatal, perinatal, or postnatal diagnosis. X-axis: the stage at which the child is referred, according to the health district; Y-axis: number of new referrals with neurodevelopmental disorders diagnosed by the Early Attention Centers of the Territorial Delegation of Health and Families.

Figure 2 shows the distribution of the cases by districts. The highest number of cases was found in the West Almeria district, closely followed by the Central Almeria district, with a significant gap between them and the West Almeria district. Additionally, Figure 2 reveals significant associations between odds ratios and confidence intervals. Based on the data, the West Almeria district was associated with the Central Almeria district and the East Almeria district for prenatal diagnosis (OR 1.45 and 2.45, respectively). It was also found that there was a correlation between perinatal diagnoses in the districts of West Almeria and Central Almeria (OR 1.29). Furthermore, a relationship was observed between prenatal diagnoses in the Central Almeria and East Almeria districts (OR 1.68).

The occurrence of risk factors contributing to diagnoses during the prenatal, perinatal, and postnatal stages is detailed in Table 2, Table 3 and Table 4. According to Table 2, chromosomal aberrations were the most common prenatal factor, accounting for 28.6% of the cases. In contrast, intrauterine physical factors were the least frequent, accounting for only 0.5%. The West Almeria district had the highest number of cases, with a maximum of 111 cases.

According to the data presented in Table 3, the most prevalent perinatal diagnosis was gestational age less than 32 weeks (25.0%). On the contrary, the incidence of newborns with hyperbilirubinemia that require exchange transfusion and dystocia or complications during childbirth that require special attention in the hours following delivery was the lowest (0.4%). The West Health district exhibited the highest number of cases, totaling 123 (44%), consistent with prenatal findings.

According to Table 4, the most prevalent problem discovered during postnatal diagnosis by neuroimaging was brain damage (36.7%). On the other hand, unspecified postnatal alterations were the least common (3.1%). The West district had the highest number of cases, 39 (40%).

Table 5 shows the correlation between the distribution of other biological factors and the health district. The most common pathology was warning signs detected in pediatric primary care teams or Early Childhood Education Centers (96.9%). The least common were unspecified biological factors (3.1%). It is worth noting that the West district had the highest number of cases (45%).

Table 6 presents the distribution of diagnoses not addressed in previous sections, classified by health districts. Of the 38 reported cases, developmental motor disorders were the most common diagnosis (34%). With medium exposure, the Central district recorded the highest number of cases (50%).

The results of the bivariate analysis for each diagnosis stage, stratified for the health district, are shown in Table 7 and Table 8. Table 7 shows the incidence of the main pathologies in prenatal, perinatal, and postnatal diagnosis in different health districts. Based on the data in Table 7, the West health district has a higher incidence of prenatal pathologies than the East district. 

Table 8 comprehensively shows the risk factors contributing to the district-wise diagnosis of prenatal, perinatal, or postnatal neurodevelopmental disorders. Upon analysis, it was observed that sociobiological risk factors, complicated pregnancies, genetic factors, information on malformations or injuries to the fetus (probable or confirmed), perinatal diagnosis of probable/possible physical or mental disability or somatic malformation, and postnatal diagnosis of probable/possible physical or mental disability, severe illness, or somatic malformation were more prevalent in the West Almeria district. On the other hand, the East Almeria district presented a lower frequency of these factors mentioned above.

The multiple binary logistic regression analysis of possible risk factors associated with neurodevelopment disorders is represented in Table 9. For the prenatal risk of developing neurodevelopmental disorders, we found a lower risk in the Central and East Almeria districts and a higher risk for male children. In this group, an OR = 0.421 (*p* < 0.001) was obtained for the variable age of referral (in years). A significant decrease in the risk of perinatal disorders was observed in the Central Almeria District and an increased risk for male children, and an OR = 0.214 (*p* < 0.001) was observed for age of referral. In addition, it can also be noted that postnatal risk was not associated with any district. However, this group showed an increased risk of learning disabilities in the postnatal stage for male children and a decreased risk according to the age of referral.

## 4. Discussion

This research aimed to determine whether living in regions with high use of agrochemicals, particularly pesticides, due to increased use in intensive agriculture is associated with a higher prevalence of neurodevelopmental disorders and their risk factors. Our results showed that chromosomal abnormalities were the most prevalent disorders among prenatal diagnoses, gestational age less than thirty-two weeks among perinatal diagnoses, and brain damage among postnatal diagnoses. Additionally, binary logistic regression analysis revealed a higher risk for children of the West Health district in prenatal and perinatal diagnoses.

Environmental exposure can occur through many sources, such as food contact materials, personal care products, contaminated drinking water, and clothing [23]. This kind of exposure can affect the population, particularly in areas with a high concentration of greenhouses. Due to favorable weather conditions, crops can be grown throughout the year in Almeria, particularly in intensive greenhouse production systems that require constant use of pesticides to prevent and manage harmful organisms that can affect plants. Areas with high pesticide use had a higher greenhouse area than areas with low pesticide use. Therefore, people living near areas with intensive agriculture, not just agricultural workers and applicators, are at increased risk of pesticide exposure. The potential neurotoxic effects of individual pesticides can be difficult to assess because farmers are often exposed to more than one pesticide. Assessing the potential neurotoxic effects of particular pesticides can be challenging because populations are frequently exposed to multiple pesticides.

When analyzing the distribution of the cases by districts (Figure 2), the highest number of cases was found in the West Almeria district, closely followed by the Central Almeria district. However, we found a significant gap between these districts and the West Almeria district. This aspect was also stated when analyzing the distribution of prenatal, perinatal, and postnatal diagnoses by health district (Table 2, Table 3 and Table 4). Furthermore, it was also observed when analyzing the distribution of other biological factors by the health district.

In recent years, the use of pesticides for crop protection has increased. As a result, exposure levels have increased for pesticide applicators, agricultural workers, and even the general population. This increase aligns with the new referrals reported by the ODAT (Figure 1). However, 2022 shows a reduction in new referrals, likely linked to under-diagnosis during the COVID-19 pandemic [33]. Our study also found that the high-use area is over 4.0 times higher than the low-use area (Table 1). This finding aligns with previous studies conducted in the same region, which estimated the pesticide use to be only 3.6 times higher [28]. This could be explained by the area with low pesticide use having a diverse range of woody crops, such as citrus, olive, almond, and fruit trees, and these crops require less pesticide application.

Additionally, our study suggests that specific learning disorders are more common and pose a greater risk to people living in areas with high levels of pesticide use than to those living in areas with low pesticide use (Table 2, Table 3, Table 4, Table 5 and Table 6). These results are in line with the conclusions of the Childhood Autism Risks from Genetics and the Environment (CHARGE) study [34]. The CHARGE study showed that exposure to agricultural pesticides during pregnancy in people living in areas with high levels of pesticide use was associated with an increased risk of certain neurodevelopmental disorders, such as ASD and developmental delay [35]. Recently, a study was conducted on Ecuadorian children living near floriculture crops. The study found that these children experienced a decline in neurobehavioral performance, especially in areas such as attention and inhibitory control, language, memory, and learning [36]. In this line, a study found that children living close to floriculture crops showed a decline in neurobehavioral performance, specifically in areas such as attention and inhibitory control, language, memory, and learning [37]. 

Our study indicates that living in areas with increased pesticide use can affect prenatal development. Among the most common prenatal alterations, genetic factors, severe disease or somatic malformation, complicated pregnancies, and sociobiological risk factors are noteworthy. Perinatal risks are mainly related to gyneco-obstetric care, while postnatal risks relate to pediatric care. Similarly, one of the most critical cohort studies for environmental pesticide exposure among children in a farming community, the CHAMACOS study, found a direct association between prenatal organophosphate exposure and childhood intelligence quotient [38]. For this reason, recent observational epidemiological studies have raised concerns about the developmental neurotoxicity caused by pesticides, particularly in children exposed prenatally or during early postnatal life [39,40,41,42].

Interestingly, according to the findings of several studies, males exhibit more pronounced neurodevelopmental effects than females [37,38]. This aspect can also be observed in the multivariate analysis of this study, where the male sex is associated with a higher risk of diagnosis in the multivariate model. According to these studies, sex differences may be influenced by biological factors such as variations in metabolism or differences in individual susceptibility. Multivariate analysis also found a correlation between the age of referral and the risk of developing neurodevelopmental disorders. In particular, the risk of developing such disorders decreased with increasing age of referral. The association with age is similar to what other clinical studies have found when evaluating this type of disorder in the pediatric population [43,44,45]. 

When evaluating the present study, it is crucial to consider its ecological nature. Although we could not access specific details on the frequency and duration of pesticide exposure in each study area, we used aggregated data to perform our analysis. This methodology enabled us to rely on agronomic data, thus increasing the understanding of the potential risks of neurodevelopmental disorders related to pesticide exposure. Moreover, it has been shown that implementing small geographical areas, called health districts, can effectively reduce ecological bias and increase homogeneity in terms of pesticide exposure [46]. Another possible limitation could be the lack of a control group. However, areas of low, medium, and high exposure are clearly defined regarding greenhouse hectares and pesticide use, establishing a clear environmental exposure pattern.

Furthermore, this study has significant strengths. It took place in an area where exposure is not limited to a single pesticide but to mixtures of pesticides due to agronomic characteristics and crop diversity. As such, it offers insight into real-world exposure scenarios to mixtures of pesticides. Additionally, as far as we know, most research investigating environmental exposure’s effects on neurodevelopment has been carried out on animals or limited to a single pesticide. 

Subsequent research efforts should conduct spatial point pattern analysis to investigate the potential correlation between environmental exposure to pesticides and neurodevelopmental health outcomes, allowing pesticide effect assessment based on area of residence.

## 5. Conclusions

The results of the present study seem to indicate a potential impact of exposure on the neurodevelopment and learning disabilities of children in areas with a high concentration of greenhouses, where pesticides are used extensively. Chromosomal abnormalities were the most prevalent disorders for prenatal diagnoses, gestational age less than thirty-two weeks was the most recurring problem among perinatal problems, and brain damage was the most common among postnatal diagnoses. Additionally, a higher risk for children of the West district in the prenatal and perinatal groups was observed. The risk of neurodevelopmental disorders showed an inverse trend with age, probably due to early detection. Concerning gender, a higher incidence of neurodevelopmental disorders was observed in male children, which may be due to genetic susceptibility. Further studies are warranted to obtain more information on the effect that long-term chronic exposure to low doses of pesticides may have on neurodevelopment in the infant population. Therefore, this study highlights the importance of thoroughly considering the potential neurotoxicity of certain chemicals, such as pesticides. Implementing stricter requirements and regulations for neurotoxicity studies before their approval could avoid the sale of products that may cause neurotoxicity and negatively impact the neurological development of children.

## Figures and Tables

**Table 1:** Demographic characteristics of the study population.

		Frequency	Rates (%)
Sex	Male	3390	70.2
	Female	1440	29.8
Age of referral	0	869	18.0
	1	799	16.5
	2	1352	28.0
	3	1067	22.1
	4	541	11.2
	5	202	4.2
District	West Almeria	1845	38.2
	Central Almeria	2015	41.7
	East Almeria	970	20.1

**Table 2:** Distribution of prenatal diagnosis by health district.

	Frequency	Rates (%)	Health District		
			West	Central	East
Family history of hearing, visual, neurological, or psychiatric disorders of possible recurrence	3	1.4	0	2	1
Twin pregnancies or higher-order multiple pregnancies	3	1.4	2	1	0
Sociobiological risk factors	5	2.3	4	1	0
Somatic malformation syndromes and congenital anomalies	44	20.0	22	19	3
CNS malformations congenital hydrocephalus	8	3.6	4	4	0
Malformations affecting other organs	11	5.0	5	3	3
Complicated pregnancies	21	9.5	13	5	3
Uterine growth retardation	9	4.1	4	4	1
Genetic factors *	33	15.0	23	6	4
Genes *	9	4.1	3	5	1
Chromosomal aberrations	63	28.6	27	30	6
Intrauterine physical factors	1	0.5	1	0	0
Other prenatal diagnosis	10	4.5	3	5	2
Total	220	100.0	111 (50%)	85 (39%)	24 (11%)

* The genes and genetic factors categories are defined separately within the biological risk factors outlined by the Early Childhood Diagnosis Organization. The genes category collects recognized anomalies such as Rett syndrome or sickle cell traits, while genetic factors encompass other genetic disorders not included in the genes section.

**Table 3:** Distribution of perinatal diagnosis by health district.

	Frequency	Rates (%)	Health District		
			West	Central	East
Low-birth-weight infant with intrauterine growth retardation with weight < P 10 for gestational age	11	3.9	7	3	1
Weight less than 1500 g	9	3.2	6	3	0
Preterm infant	25	8.9	15	8	2
Gestational age < 37 weeks	11	3.9	2	6	3
Gestational age < 32 weeks	70	25.0	19	38	13
Gestational age < 28 weeks	42	15.0	12	17	13
Newborn with Apgar <3 at 1 min or <7 at 5	15	5.4	6	4	5
Newborns with mechanical ventilation for more than 24 h	3	1.1	0	3	0
Newborn with neonatal distress and other respiratory dysfunction	41	14.6	26	12	3
Severe asphyxia	10	3.6	5	2	3
Newborn with hyperbilirubinemia requiring exchange transfusion	1	0.4	1	0	0
Neonatal convulsions	7	2.5	5	2	0
Persistent neurological dysfunction (more than 7 days)	11	3.9	6	4	1
Sepsis, meningitis, or neonatal encephalitis	9	3.2	6	0	3
Dystocia or problems in a delivery requiring special attention in the hours following delivery	1	0.4	1	0	0
Other	14	5.0	6	2	6
Total	280	100.0	123 (44%)	104 (37%)	53 (19%)

**Table 4:** Distribution of postnatal diagnosis by the health district.

	Frequency	Rates (%)	Health District		
			West	Central	East
Postnatal infections of the CNS	4	4.1	1	3	0
Accidents and trauma with neurological, motor, or sensory sequelae	4	4.1	0	0	4
Chronic illnesses with a complicated course leading to continuous health care and hospitalization	13	13.3	4	5	4
Epilepsy	13	13.3	5	6	2
Pondoestatural retardation	8	8.2	5	2	1
Chronic endocrinological and metabolic disorders	6	6.1	4	1	1
Brain damage evidenced by neuro-imaging	36	36.7	13	18	5
Hearing loss detected in early detection programs (Otoacoustic emissions or Auditory Potential Hearing Impairment)	4	4.1	1	1	2
Visual disturbances	7	7.1	5	1	1
Other	3	3.1	1	0	2
Total	98	100.0	39 (40%)	37 (38%)	22 (22%)

**Table 5:** Distribution of other biological factors by the health district.

	Frequency	Rates (%)	Health District		
			West	Central	East
Alert signs detected in Primary Care Paediatric Teams or Early Childhood Education Centres	63	96.9	28	26	9
Others	2	3.1	1	1	0
Total	65	100.0	29 (45%)	27 (42%)	9 (13%)

**Table 6:** Distribution of diagnoses by health district.

	Frequency	Health District		
		West	Central	East
Developmental motor disorders	38	13 (34%)	19 (50%)	6 (16%)
Cerebral palsy/cerebral motor disorder	60	22 (37%)	28 (47%)	10 (17%)
Disorders of spinal origin	6	3 (50%)	3 (50%)	0 (0%)
Disorders of peripheral origin	4	4 (100%)	0 (0%)	0 (0%)

**Table 7:** Comparison by the district for the main pathologies classified by prenatal, perinatal, and postnatal diagnosis.

Diagnosis	District	Frequencies	Rates (^0^/_00_)	Chi-Square	*p*-Value
Prenatal diagnosis	West Almeria	112	2.26	10.79	0.004
	Central Almeria	84	1.72		
	East Almeria	24	1.11		
Perinatal diagnosis	West Almeria	123	2.48	1.48	0.470
	Central Almeria	104	2.13		
	East Almeria	53	2.45		
Postnatal diagnosis	West Almeria	40	0.80	0.56	0.750
	Central Almeria	36	0.73		
	East Almeria	22	1.02		

**Table 8:** District-specific prenatal, perinatal, and postnatal diagnosis rates.

Diagnosis	District	Frequencies	Rates (^0^/_00_)	Chi-Square	*p*-Value
Sociobiological risk factors	West Almeria	5	0.10	13.50	0.001
	Central Almeria	0	0.00		
	East Almeria	0	0.00		
Complicated pregnancies	West Almeria	13	0.26	8.85	0.010
	Central Almeria	5	0.10		
	East District	3	0.13		
Genetic factors	West Almeria	26	0.52	20.00	<0.001
	Central Almeria	11	0.22		
	East Almeria	5	0.23		
Information on malformations or injuries to the fetus (probable or confirmed)	West Almeria	6	0.12	173.90	<0.001
	Central Almeria	1	0.02		
	East Almeria	0	0.00		
Perinatal diagnosis of probable/possible physical or mental disability or somatic malformation	West Almeria	5	0.10	169.70	<0.001
	Central Almeria	2	0.04		
	East Almeria	0	0.00		
Postnatal diagnosis of probable/possible physical or mental disability, severe illness, or somatic malformation	West Almeria	14	0.28	36.37	<0.001
	Central Almeria	3	0.06		
	East Almeria	0	0.00		

**Table 9:** Binary logistic regression analysis of prenatal, perinatal, and postnatal risk.

		Exp(B)	95% CI	*p*-Value
Prenatal risk	Health district (West Almeria)	Ref.		
	Health district (Central Almeria)	0.659	0.489–0.889	0.006
	Health district (East Almeria)	0.451	0.285–0.713	<0.001
	Gender (male)	1.489	1.120–1.979	0.008
	Age at referral (in years)	0.423	0.368–0.485	<0.001
Perinatal risk	Health district (West Almeria)	Ref.		
	Health district (Central Almeria)	0.735	0.550–0.983	0.038
	Health district (East Almeria)	1.011	0.702–1.456	0.951
	Gender (male)	1.510	1.159–1.968	0.002
	Age at referral (in years)	0.214	0.178–0.258	<0.001
Postnatal risk	Gender (male)	2.104	1.400–3.164	<0.001
	Age at referral (in years)	0.626	0.530–0.739	<0.001

The regression model was adjusted for the health district (0: West; 1: Central; 2: East), gender (0: female; 1: male), and age of referral. The Nagelkerke R^2^ was 0.16, 0.38, and 0.06, respectively, and the *p*-value for the Hosmer–Lemeshow test was lower than 0.002, 0.026, and 0.001, respectively.

## Data Availability

The data presented in this study are available on request from the corresponding author.
